# Root pH variation of herbaceous plants among plant functional groups in response to climate and soil gradients on the Tibetan alpine grasslands

**DOI:** 10.1002/ece3.70060

**Published:** 2024-07-21

**Authors:** Lirong Zhao, Bo Pang, Jiangtao Hong, Xingxing Ma, Ziyin Du, Xiaodan Wang

**Affiliations:** ^1^ Institute of Mountain Hazards and Environment Chinese Academy of Sciences Chengdu China; ^2^ University of Chinese Academy of Sciences Beijing China; ^3^ College of Urban and Environment Sciences Shanxi Normal University Linfen China; ^4^ School of Land and Resources China West Normal University Nanchong China

**Keywords:** alpine ecosystems, functional traits, soil pH, transect, water availability

## Abstract

Plant pH is an emerging functional trait that plays important roles in physiological processes and nutrient cycling. However, how root pH varies among plant functional groups (PFGs) and the regulatory factors on a large scale remain unclear. Therefore, we quantified root pH variation of herbaceous plants in four PFGs from 20 sites on the Tibetan Plateau along a 1600 km transect and explored the correlations between root pH and different PFGs, climate and soil conditions. The results showed that the root pH of herbaceous plants was slightly acidic (6.46 ± 0.05). Grasses had the highest root pH (6.91 ± 0.10) across all functional groups (*p* < .05), whereas legumes had the lowest (5.90 ± 0.08; *p* < .05). The root pH decreased with mean annual precipitation, aridity index, soil water content and soil stress coefficient, whereas the significant positive correlation with soil pH. PFGs, climate and soil explained 5.39, 11.15 and 24.94% of the root pH variance, respectively. This study provided a comprehensive analysis of root pH patterns in herbaceous plants over a large spatial scale. Root pH was controlled by the combined influence of PFGs, climate and soil properties, with moisture status being the main influential factor. In contrast to the leaf pH, the root pH of herbaceous plants is strongly affected by the soil pH along environmental gradients. Our findings provide new insights into root functional traits and survival strategies of herbaceous plants in alpine ecosystems.

## Graphical abstract

In this study, we conducted a comprehensive analysis of herbaceous root pH patterns on a large‐scale space. The main ecosystem types included alpine meadows, alpine grasslands, and alpine deserts with a total of 25 plant species, of which the main species were *Kobresia pygmaea*, *Kobresia littledalei*, *Stipa purpurea*, *Carex moorcroftii*, *Stipa glareosa* and *Ceratoides compacta*.

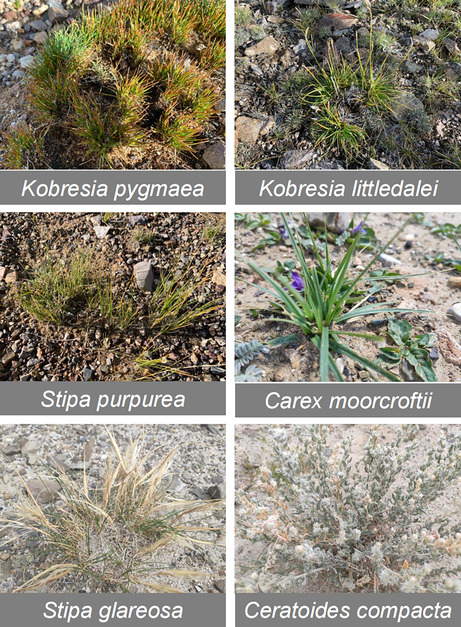

## 1 INTRODUCTION

Plant functional traits are broadly employed to predict changes in ecosystem functioning driven by differences or shifts in environmental gradients (Arndt, 2006; Chapin et al., 1993; Li et al., 2022; Schellberg & Pontes, 2012; Wright et al., 2004). Plant pH has recently been introduced as a new plant functional trait for its predictive power in plant physiological processes and ecosystem carbon cycling processes, such as herbivory (Cornelissen et al., 2006), litter decomposition (Freschet et al., 2012; Tao et al., 2019) and mycorrhizal symbiosis (Cornelissen et al., 2006). Plant pH is jointly regulated by a highly environmentally sensitive apoplastic pH and relatively stable intracellular pH (Frohnmeyer et al., 1998; Kurkdjian & Guern, 1989; Liu et al., 2019). The pH of plant tissue reflects the overall aspect of apoplastic and intracellular pH and is more likely regulated by the changes in apoplastic pH in a changing environment because of the relatively stable status of intracellular pH (Epstein & Bloom, 2005; Frohnmeyer et al., 1998).

Compared to the measurement of apoplastic and cellular pH, leaf/root pH is a fast quantitative trait which is cost effective and widely used in woody and herbaceous plants (Cornelissen et al., 2011; Liu et al., 2019; Sattelmacher et al., 1998). Plant tissue apoplastic pH can be regulated by multiple environmental factors such as vapour pressure deficit, water stress, light intensity and hypoxia (Felle, 2001; Felle & Hanstein, 2002; Hedrich et al., 2001; Karuppanapandian et al., 2017). Considering the key role of root pH as a signal controlling apical growth, plant hormone responses and other physiological activities in plants, more efforts should be made to investigate root pH in different ecosystems (Scott & Allen, 1999).

Plant nutrient trait concentrations (C and N concentrations and C:N ratios), physical properties (toughness) and recalcitrant components (tannin and lignin content) have all been linked to interspecific variations in litter decomposability (García‐Palacios et al., 2016; Parton et al., 2007; Yue et al., 2016). However, leaf and root pH have recently been shown to be useful predictors of litter decomposition where plant litter pH is positively correlated with litter decomposition rates in subarctic plant species (Cornelissen et al., 2006; Freschet et al., 2012; Tao et al., 2019). This may be because higher plant pH results in a higher base cation content (Liu et al., 2019), beneficial for the decomposer activity (Cornelissen & Thompson, 1997; Makkonen et al., 2012; Tao et al., 2019). In contrast, acidic plant tissue tends to be rich in recalcitrant organic acids that can inhibit decomposition (Hättenschwiler & Vitousek, 2000; Mcclain et al., 1997). Thus, interspecific variation in plant pH is an important driver of litter decomposition rates based on the strong ‘afterlife’ effects of these traits on litter decomposability (Cornelissen, 1996; Tao et al., 2019), which in turn can alter the pH of soil organic matter and carbon cycling processes.

Environmental and biological factors are two important factors regulating variations in plant pH. Generally, drought stress (low precipitation) can increase leaf pH by influencing the transport of abscisic acid to leaf apoplasts and improving intracellular Ca^2+^ accumulation (Felle, 2001). Under excess light conditions, plants can induce a protective mechanism by reducing the thylakoid membrane pH to reduce or prevent leaf damage (Niyogi et al., 2005; Wraight et al., 1972). However, soil pH had no significant influence on foliar pH in 64 herbaceous species across northern China (Liu et al., 2019) and 23 herbaceous species (Cornelissen et al., 2011) in temperate European grasslands. Biological characteristics tend to have a more notable effect on leaf pH variation. Studies on foliar pH variations based on changes in the surrounding environment and biological factors have improved our knowledge of the stress resistance and resilience of plants in changing environments. However, previous studies have mainly focused on leaf or litter pH. The variability in root pH under varying environmental conditions and among different functional groups remains poorly addressed (Cornelissen et al., 2011; Luo et al., 2021; Tao et al., 2019). In addition, current knowledge has mainly been obtained from pot culture and local‐scale investigations, with little research yet conducted concerning the variability of biogeographical root pH patterns at large scales (Freschet et al., 2012).

Tibetan alpine grasslands are considered an ideal ecosystem for exploring plant functional trait responses to environmental gradients because they are less disturbed by human activity than other areas globally. In the past, there have been more studies on the variation of soil pH in different environmental conditions in the Tibetan Plateau (Dai et al., 2023; Sun et al., 2023). However, the variability of root pH in diverse environmental conditions and across various species is still inadequately explored. Here, we conducted a systematic survey of the root pH of 264 plant samples from 25 species and eight families that covered common species in the Tibetan grasslands of China. Our objectives were to (1) quantify root pH variations across different plant functional groups (PFGs) (grasses, sedges, legumes and forbs) and (2) clarify root pH changes along climatic and soil gradients in alpine grasslands.

## 2 MATERIALS AND METHODS

### 2.1 Study area

This study was conducted based on an investigation of 20 sampling sites (79.75 °E–94.62 °E, 29.64 °N–33.43 °N) in Tibetan grasslands (Table 1). The sampling sites were selected according to grassland type (alpine meadow, alpine steppe and alpine desert) and environmental gradients (Figure S1). The sampling sites were located in 11 counties (Bayi, Maizhokunggar, Lhunzhub, Damxung, Baingoin, Xainza, Nyima, Gerze, Geji, Gar and Rutog). To reduce the influence of human disturbance on plant and soil physical and chemical properties, we selected sampling sites far away from cities, towns, villages and main roads. In addition, all the sample sites were selected on flat ground to minimise topographic variation. The five alpine meadow sites were dominated by *Kobresia pygmaea* and *K. littledalei*. The 11 alpine steppe sites were dominated by *Stipa purpurea* and *S. glareosa* and the accompanying species included *Carex moorcroftii*, *Leontopodium nanum*, *Oxytropis glacialis* and *O. microphylla*. The largest number of sampling sites in the alpine steppe matched well with the maximum area of this type of grassland in the Tibetan Autonomous Region. The four alpine desert sites were dominated by *Stipa glareosa* and *Ceratoides compacta* (Figure S1). The sampling sites covered all three grassland types and common species and represented the general distribution of Tibetan grassland vegetation types. Additional information on vegetation conditions at each sampling site is shown in Table S1. The major soil type of the alpine grasslands is Haplic Xerosol, according to the World Reference Base for Soil Resources (Lu et al., 2004).

**TABLE 1:** Geographic information and vegetation characteristics of sampling sites.

Site	Latitude (°N)	Longitude (°E)	Altitude (mm)	Ecosystem	Species	Root pH		
						Mean	SE	*n*
Bayi	29.64	94.62	4456	Alpine meadow	*Kobresia humilis, Carex moorcroftii, Potentilla bifurca, Saussurea leontodontoides, Polygonum capitatum*	5.61	0.16	15
Maizhokunggar	29.82	92.36	4911	Alpine meadow	*Kobresia littledalei, Kobresia pygmaea, Carex moorcroftii*	6.00	0.14	9
Lhunzhub	30.42	91.02	4249	Alpine meadow	*Elymus nutans, Poa litwinowiana, Kobresia pygmaea, Carex moorcroftii, Potentilla bifurca*	6.22	0.14	15
Damxung	30.15	91.24	4314	Alpine meadow	*Elymus nutans, Poa litwinowiana, Kobresia pygmaea, Carex moorcroftii, Chenopodium tibeticum*	6.27	0.18	15
Baingoin	31.39	90.78	4621	Alpine steppe	*Stipa purpurea, Kobresia humilis, Leontopodium nanum, Saussurea tibetica*	6.60	0.12	12
Baingoin	31.09	90.61	4811	Alpine meadow	*Kobresia pygmaea, Stipa purpurea, Leontopodium nanum, Saussurea tibetica, Potentilla bifurca*	6.55	0.11	15
Baingoin	31.62	89.50	4679	Alpine steppe	*Leontopodium nanum, Kobresia humilis, Saussurea tibetica, Potentilla bifurca, Stipa purpurea*	6.18	0.08	15
Xainza	30.98	88.76	4969	Alpine steppe	*Stipa purpurea, Kobresia pygmaea, Oxytropis glacialis, Astragalus confertus, Leontopodium nanum*	6.26	0.16	15
Nyima	31.50	87.49	4652	Alpine steppe	*Leontopodium nanum, Stipa purpurea, Oxytropis microphylla, Ptilotricum canescens*	6.74	0.09	12
Nyima	31.93	86.50	4726	Alpine steppe	*Astragalus confertus, Potentilla bifurca, Stipa purpurea, Ptilotricum canescens*	6.49	0.12	12
Nyima	32.01	85.43	4869	Alpine steppe	*Stipa purpurea, Astragalus confertus, Leontopodium nanum, Saussurea tibetica*	6.93	0.14	12
Gerze	32.31	83.77	4384	Alpine steppe	*Stipa glareosa, Oxytropis microphylla, Leontopodium nanum, Potentilla bifurca, Aster hispidus*	6.96	0.11	15
Gerze	32.29	84.13	4434	Alpine steppe	*Poa litwinowiana, Stipa glareosa, Oxytropis glacialis, Potentilla bifurca*	6.67	0.15	12
Geji	32.31	82.41	4618	Alpine steppe	*Poa litwinowiana, Stipa glareosa, Oxytropis glacialis, Oxytropis microphylla*	5.86	0.29	12
Geji	32.08	81.82	4606	Alpine steppe	*Artemisia demissa, Stipa glareosa, Oxytropis microphylla, Potentilla bifurca*	6.64	0.27	12
Geji	32.33	81.21	4542	Alpine steppe	*Artemisia wellbyi, Stipa glareosa, Astragalus confertus*	6.47	0.29	9
Gar	32.35	80.71	4637	Alpine desert	*Stipa glareosa, Oxytropis microphylla, Aster hispidus, Ajania fruticulosa*	6.19	0.25	12
Rutog	33.43	79.75	4269	Alpine desert	*Artemisia wellbyi, Stipa glareosa, Ajania fruticulosa, Suaeda glauca, Ceratoides latens*	7.49	0.16	15
Rutog	32.84	79.78	4444	Alpine desert	*Stipa glareosa, Oxytropis microphylla, Christolea crassifolia, Ceratoides latens, Ptilotricum canescens*	6.72	0.19	15
Rutog	32.56	80.06	4416	Alpine desert	*Stipa glareosa, Oxytropis microphylla, Christolea crassifolia, Ajania fruticulosa, Ptilotricum canescens*	6.24	0.21	15

*Note*: Means and standard errors of root pH were shown for each site and *n* represents the sample size.

### 2.2 Soil and climate data

To investigate the variability in herbaceous root pH along soil and climatic condition gradients, we examined four climatic and eight soil variables. Climate factors included mean annual temperature (MAT, °C), mean annual precipitation (MAP, mm), solar radiation (SR, MJ m^2^/day) and aridity index (AI). The soil physical and chemical properties included soil pH, soil water content (SWC), soil cation exchange capacity (CEC), soil stress coefficient (*K*_soil_, %: a smaller *K*_soil_ denotes less soil moisture content and more drought) and soil Ca, K, Mg and Na concentrations. Soil pH, SWC, CEC and alkaline nutrients were measured in the laboratory. *K*_soil_ and AI were extracted from the database of the Consultative Group on International Agricultural Research‐Consortium for Spatial Information (CGIAR‐CSI; http://www.cgiar‐csi.org), whereas MAT, MAP and SR were obtained from WorldClim (version 2.0; Fick & Hijmans, 2017).

### 2.3 Sampling and measurement

Twenty sites were selected in August 2020 and at each site, three 100 × 100 m quadrats were established. Dominant and common species were selected and sampled from each site. However, at some species‐poor sites, as few as three species were collected (Table 1). A soil core (20 cm in length, 20 cm in width and 30 cm in depth) containing the target species was collected using a spade in the quadrat. All the species were sampled from mature and healthy individuals. Individual plants with obvious evidence of biotic alterations (e.g., bird droppings or disease) or mechanical damage were not sampled. Because of the small individual size of herbaceous plants on the Tibetan Plateau, the replicates consisted of multiple individuals of each species. Three replicates were collected from each species and each replicate contained at least 10 individuals and 15 g of living roots. The live roots were identified based on their consistency and colour (Vogt & Persson, 1991).

All species were divided into four functional groups: grasses, sedges, legumes and forbs (Hong et al., 2015; Mamolos et al., 2005; Song et al., 2012; Tables 1 and S1). Overall, our database consisted of 25 species (264 individuals) from 8 families and 19 genera, including four grass species (66 individuals), four sedges (33 individuals), three legumes species (48 individuals) and 14 forbs (117 individuals; Table 1). Among them, only 13 species were measured at multiple (3–9) sites, whereas each of the other species was measured only at one or two sites. The root pH variation of the same species along soil and climate gradients was explored to further verify the effect of environmental gradients on intraspecific variation.

Fresh root samples were washed using deionised water, dried quickly with paper towels, assembled in Ziplock bags and frozen in a refrigerator at −24°C. The samples were then taken back to the laboratory, ground manually and then extracted in deionised water (1:10; Liu et al., 2019). The root–water mixture was shaken at 250 rpm for 1 h and subsequently centrifuged until solid–liquid separation (Cornelissen et al., 2011). In each site, soil samples were collected from the centre of each quadrat (1 × 1 m) and thoroughly mixed. Soil samples were air‐dried and passed through a 2 mm sieve to remove plant litter, roots and gravel. Soil pH was measured potentiometrically at a soil‐water suspension mass ratio of 1:2.5 (Bao, 2005). Both the root and soil pH were measured using a precise pH meter. Soil water content (%) was measured after drying at 105°C for 48 h. The CEC was determined by percolating soil columns with 1 M ammonium acetate buffered at pH 7 (Metson, 1956). Soil Ca, K, Mg and Na concentrations were determined using ICP‐OES (iCAP‐7200; Thermo Fisher Scientific, USA). The soil and climate variables at each sampling site are listed in Table S2.

### 2.4 Data analysis

We examined the effects of PFGs on root pH using one‐way analysis of variance (ANOVA). Linear regressions were used to explore the relationships among root pH, geographical factors (latitude, longitude and altitude), climatic factors (MAP, MAT, AI and SR) and edaphic variables (*K*_soil_, pH, WC, CEC, Ca, K, Mg and Na). Variations in plant root pH were partitioned among three explanatory variable groups (PFGs, climate and soil) using partial regression analysis with redundancy analysis. Forward selection was performed for each variable and root pH. MAP, MAT, AI, SR, *K*_soil_, pH, WC, CEC, Ca, K, Mg and Na were treated as independent variables in the forward regression model. Variables that did not notably contribute to root pH were excluded from subsequent variation‐partitioning analyses. This analysis was conducted using ‘vegan’ in the R software package (Oksanen et al., 2022; R. C. Team, 2022). All statistical analyses were performed using R 3.4.3 and SPSS 16.0. The figures were plotted using SigmaPlot 11 and R 3.4.3.

## 3 RESULTS

### 3.1 Root pH variations across different PFGs

On the Tibetan Plateau the root pH of the herbaceous plants was mildly acidic (6.46 ± 0.05; Table 2). The root pH of the grasses (6.91 ± 0.10) was significantly higher than that of the sedges (6.40 ± 0.08), legumes (5.90 ± 0.08) and forbs (6.45 ± 0.06; *p* < .05; Table 2). Across the three types of alpine grasslands, the pH of the herbaceous plants generally increased from the alpine meadows to the alpine steppes and deserts (Table 2). The root pH of the monocotyledonous plants was higher than that of the dicotyledonous plants (Table 2).

**TABLE 2:** Variation in root pH between different plant functional groups (PFGs), phylogenies and ecosystems.

	Alpine meadow			Alpine steppe			Alpine desert			All sites		
	Mean	SE	*n*	Mean	SE	*n*	Mean	SE	*n*	Mean	SE	*n*
Functional group												
Grasses	6.23Ac	0.22	15	6.95Ab	0.10	39	7.60Aa	0.11	12	6.91A	0.10	66
Sedges	6.34Ab	0.08	30	6.99Aa	0.10	3	–	–	–	6.40B	0.08	33
Legumes	–	–	–	5.90Ba	0.09	39	5.90Ca	0.21	9	5.90C	0.08	48
Forbs	5.84Bb	0.13	24	6.63Aa	0.05	57	6.57Ba	0.14	36	6.45B	0.06	117
Phylogeny												
Monocotyledon	6.30Ac	0.09	45	6.95Ab	0.09	42	7.60Aa	0.11	12	6.74A	0.07	99
Dicotyledon	5.84Bb	0.13	24	6.34Ba	0.06	96	6.44Ba	0.12	45	6.29B	0.05	165
Overall species	6.14b	0.08	69	6.52a	0.06	138	6.68a	0.12	57	6.46	0.05	264

*Note*: Differences in root pH between the PFGs, phylogenies and ecosystem were compared using ANOVA and independent *t*‐test. Different letters indicate statistically significant differences of root pH at the 0.05 level. The lowercase letters indicate the difference in root pH among different ecosystems for the same PFGs and phylogenies (horizontally), while the capital letters denote the differences in root pH among different PFGs and phylogenies in the same ecosystem (vertically). *n* represents the size of the sample.

### 3.2 Root pH changes along the environment gradients

**FIGURE 1:**
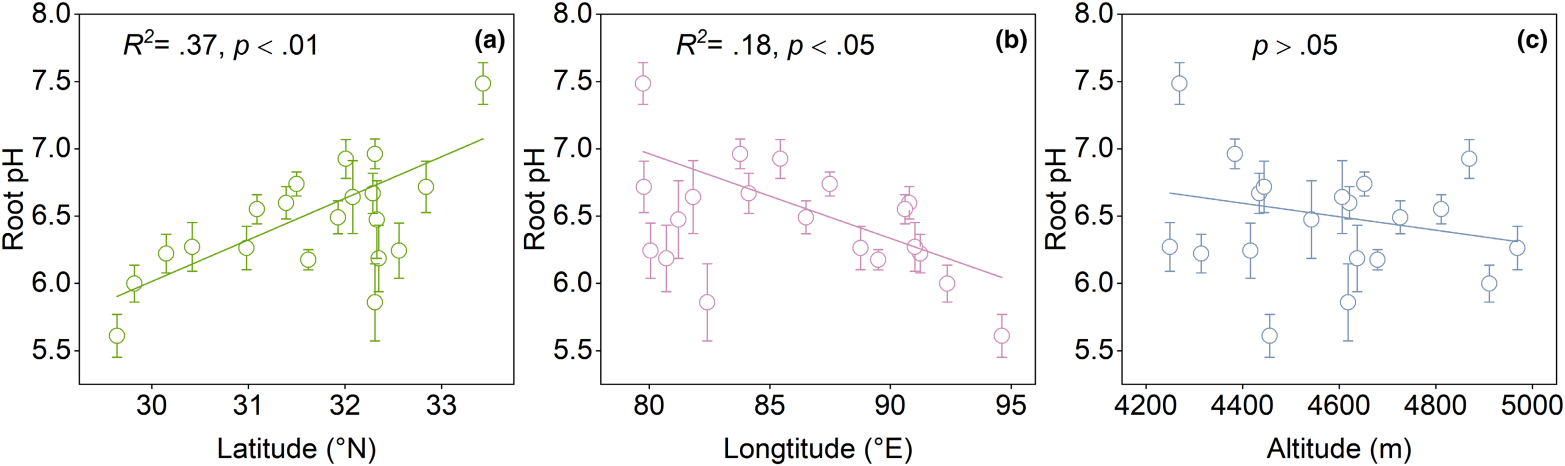
Root pH in relation to latitude, longitude and altitude. Points and error bars show the means and standard errors for each site.

For all species pooled, root pH decreased with decreasing latitude and increasing longitude (*p* < .05; Figure 1a,b). Altitude had no significant influence on root pH along the gradient (*p* > .05; Figure 1c).

**FIGURE 2:**
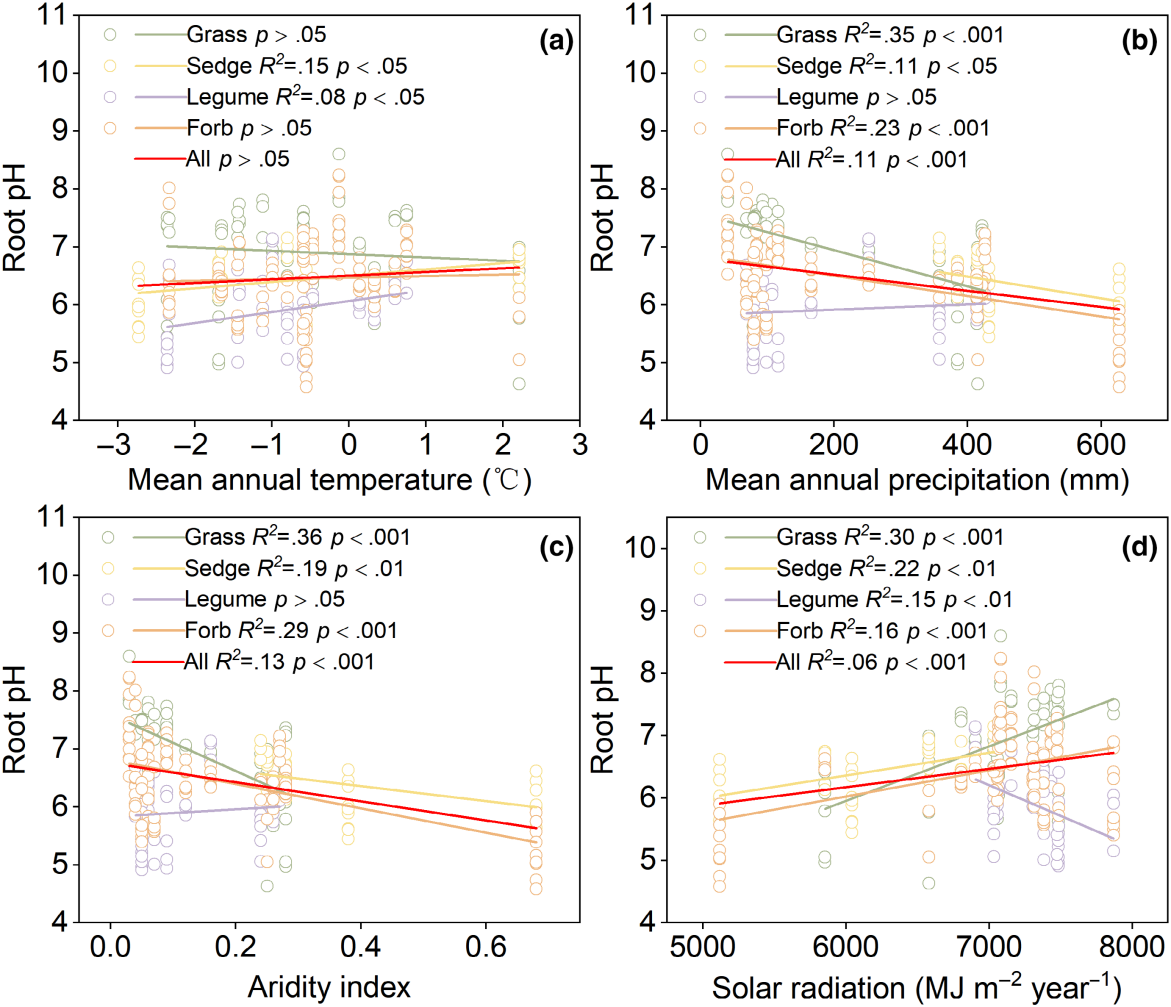
Root pH in relation to climate variables.

There was no significant relationship between root pH and MAT in any species (*p* > .05; Figure 2a). The root pH significantly decreased with increasing MAP and AI (Figure 2b,c). The root pH of grasses, sedges and forbs decreased with increasing MAP (*p* < .05) and AI (*p* < .05). However, the root pH of legumes showed no significant relationship with MAP or AI (*p* > .05). The root pH increased with solar radiation across all plants, grasses, sedges and forbs (*p* < .01); however, that of legumes showed a decreasing trend with increasing solar radiation (*p* < .01; Figure 2d).

**FIGURE 3:**
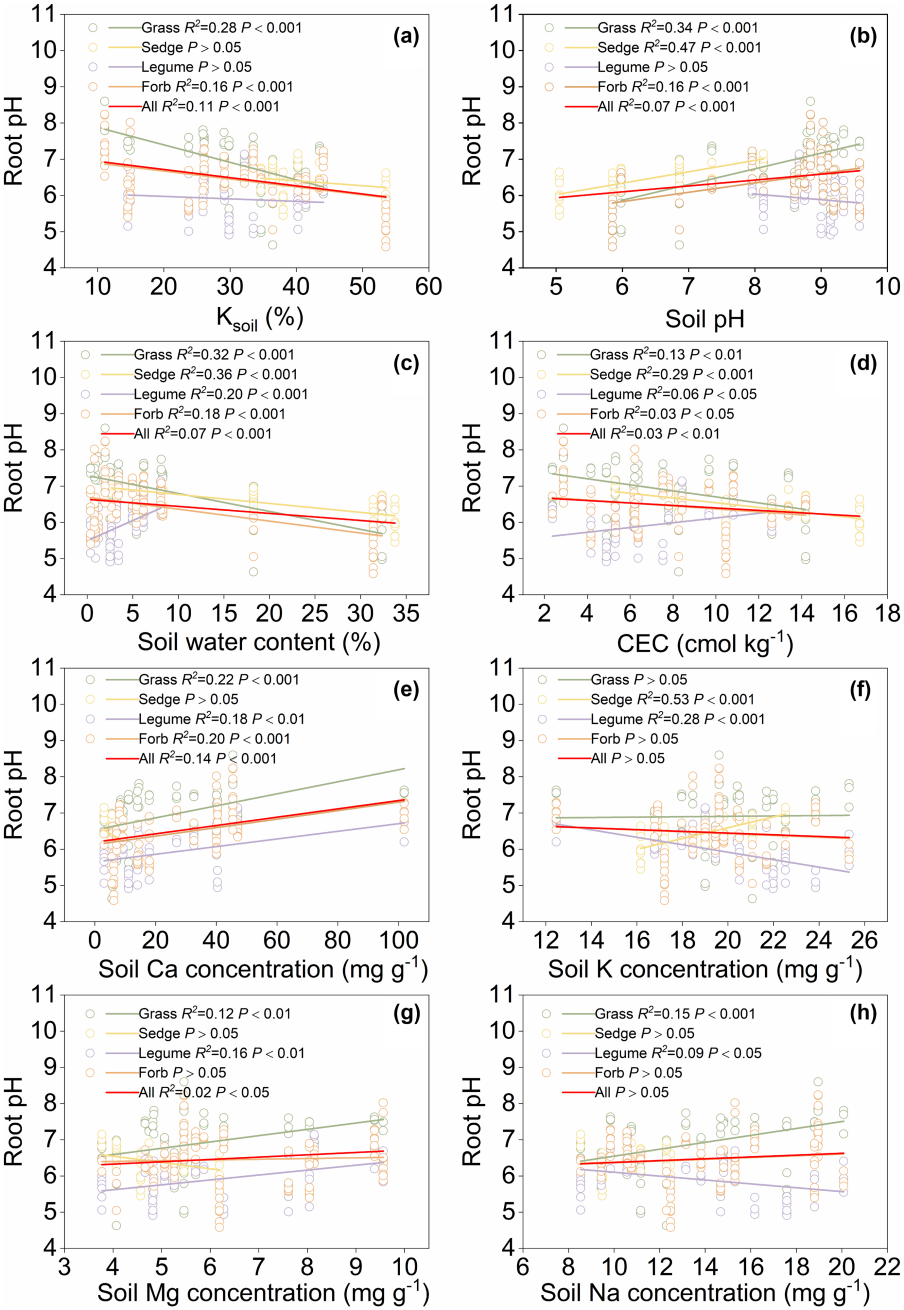
Changes of root pH along the soil gradients. *K*_soil_, soil water stress coefficient; CEC, cation exchange capacity.

The root pH of herbaceous plants showed a significant negative correlation with *K*_soil_, water content and CEC content (*p* < .01), whereas it exhibited an increasing trend with soil pH (*p* < .001; Figure 3a–d). In general, the root pH of grasses, sedges and forbs showed similar patterns to the variations in soil physicochemical properties. Legumes showed no significant correlation with *K*_soil_ and soil pH (*p* > .05; Figure 3a,b). Soil Ca and Mg concentrations were positively correlated with root pH (*p* < .05), whereas soil K and Na concentrations had no significant effect on root pH across any species (*p* > .05; Figure 3e–h). In terms of functional groups, soil Ca had the most significant promoting effect on the root pH of grasses and forbs, whereas soil K also had a significant promoting effect on the root pH of sedges (Figure 3e–h).

### 3.3 Relative effects of climate, soil and PFGs

**FIGURE 4:**
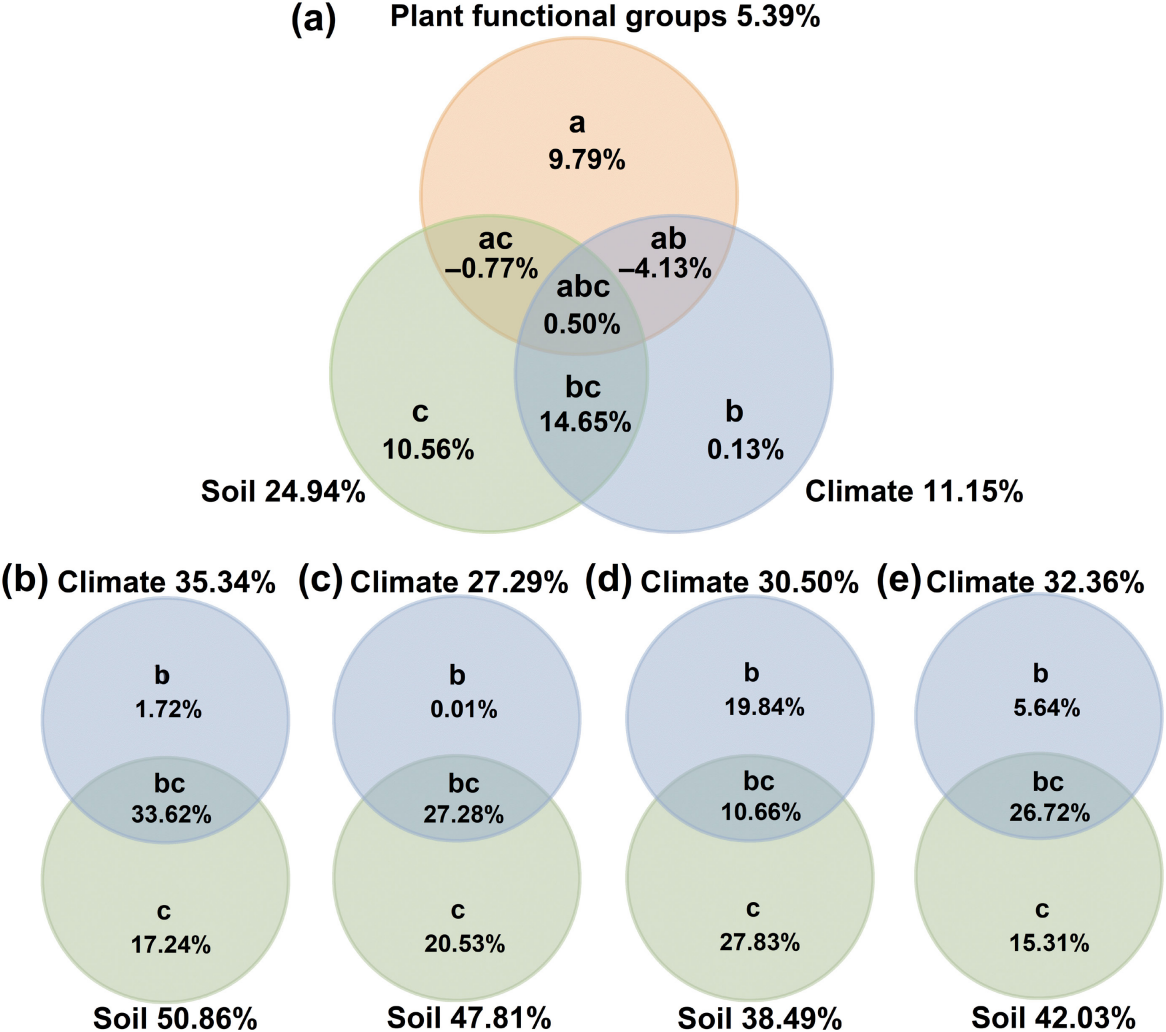
Variation partitioning (*R* ^2^) of plant functional groups (PFGs), climate and soil in accounting for the variance in root pH. In the partial general linear model (GLM), the symbols a, b and c represent the independent effects of PFT, climate and soil, respectively; ab, bc and ac are, respectively, the interactive effects between PFT and climate, climate and soil and PFT and soil; and abc represents the interactive effects among the three different factors. Within each PFG, the symbols a and b represent the independent effects of climate and soil and ab represents the interactive effects between the two factors.

Partial regression analysis revealed that three explanatory variable factors were used in the model: PFGs (grasses, sedges, legumes and forbs), climate (MAT, MAP, AI and SR) and soil (*K*_soil_, pH, WC, CEC, Ca, K, Mg and Na). These three factors together accounted for 30.73% of the variation in root pH, of which PFGs, climate and soil independently explained 5.39, 11.15 and 24.94%, respectively. The intersection of PFGs and climate (ab), PFGs and soil (ac), climate and soil (bc) and PFGs, climate and soil (abc) explained −4.13, −0.77, 14.65 and 0.50% of the variance, respectively (Figure 4a). Within each PFG, climate accounted for 35.34, 27.29, 30.50 and 32.36% of the root pH variance in grasses, sedges, legumes and forbs, respectively, while soil accounted for 50.86, 47.81, 38.49 and 42.03% of the root pH variance in grasses, sedges, legumes and forbs, respectively (Figure 4b–e).

## 4 DISCUSSION

Here, large‐scale biogeographical patterns of root pH and their driving factors in herbaceous plants were investigated. The root pH was controlled by the combined influence of PFG, climate and soil properties. Grasses had the highest root pH, whereas legumes had the lowest among the four PFGs. Moisture status, such as MAP, AI, WC and *K*_soil_, was a stronger regulator driving root pH variability than temperature along environmental gradients. A strong positive relationship was observed between soil and root pH across all herbaceous species on the Tibetan Plateau, inconsistent with previous results that found that soil pH had no significant influence on leaf pH (Cornelissen et al., 2011; Liu et al., 2019).

### 4.1 Root pH variation across PFGs

Differences in plant pH between different PFGs have been observed in typical subarctic terrestrial ecosystems (Cornelissen et al., 2006). Relatively high leaf pH was observed in graminoids and ferns, whereas sedges, rushes and ecto‐mycorrhizal plants had lower foliar pH. The root pH of common herbaceous plants varied widely across the Tibetan Plateau (ranging from 4.58 to 8.60). In biogeographical history, species originated from humid and warm areas, then dispersed to dry and cold inland regions and plant pH evolved accordingly to adapt to the changing external environment (Brown et al., 1998; Smith et al., 2010). On the Tibetan Plateau, sedges, such as *Kobresia pygmaea* and *K. littledalei*, are the dominant species in alpine meadows, whereas in alpine steppes and deserts the dominant species gradually turn to grasses, such as *Stipa purpurea* and *S. glareosa*, with an increasing aridity gradient. The dispersal of dominant species to the arid inland was accompanied by an increasing root pH across all sites (sedges: 6.40 ± 0.08, grasses: 6.91 ± 0.10). In addition, grasses are generally rich in secondary metabolites, such as tannic acids and lignin (Hong et al., 2021), which could result in higher tissue pH. Lastly, grass species generally have clustered root systems with higher root length densities, higher specific root areas and good root elongation (Li et al., 2006). This improves the absorption capacity and transportation of Ca and Mg through the apoplast and therefore these base cations may accumulate for monocotyledonous plants in alpine steppe and desert environments (Luo et al., 2016; Figure S1). The more positive the metal in the root, the more hydrogen ions might be replaced, which could result in a higher root pH (Liu et al., 2019).

Although Cornelissen et al. (2006) found that nitrogen‐fixing plants had a relatively higher foliar pH than other functional groups, the root pH of legume species in this study was significantly lower than that of the other three functional groups. This may be because the physiological and ecological characteristics of these two organs are affected by different regulatory mechanisms. Leaves belong to the ‘metabolic’ component, while roots belong to the ‘structural’ component (Hong et al., 2015). In addition, legume species on the Tibetan Plateau have developed specific axial root systems with finer roots distributed in deeper soil layers (Hong et al., 2018), which can help avoid excessive basic cation accumulation and support plants in absorbing water from deeper soils. Finally, legume species invest a larger fraction of biomass to roots – when compared with leaves and shoots – causing ‘diluting effects’ on basic cation concentrations and resulting in a low root pH for those species (Luo et al., 2016).

### 4.2 Root pH changes along the environmental gradients

Moisture status, such as MAP, AI, WC and *K*_soil_, was a stronger regulator driving the root pH variability than the temperature along the climate and soil gradients (Figures 3 and 4; Table 2) with a positive correlation between these moisture factors (Table S2). The root pH of herbaceous plants increased with enhanced drought stress in Tibetan grasslands. Generally, pH is regarded as a drought stress signal or message in situations where the roots communicate with the leaves and stems, based on the fact that the roots are in contact with dry soil (Davies & Zhang, 1991; Wilkinson, 1999). One way plants do this is by producing and transporting the stress phytohormone abscisic acid to the apoplast by increasing the apoplastic pH which, upon arrival at the stomata, initiates their closure (Felle, 2001). In addition, to improve water absorption capacity, plants can reduce their water potential by increasing the amount of base cations under water stress, which concurrently increases the root pH (Brady et al., 2000; Liu et al., 2019; Schurr et al., 1992). In contrast, in areas with abundant rainfall, the proximity of the soil to the saturated water content leads to hypoxia in the plant roots. In alpine meadow sites, soil water content could be over 30%, close to or up to saturated soil water content, thus the anaerobic environment causes the stress of hypoxia on plant roots (Felle, 2001). It has been repeatedly proven in a variety of plant cells that hypoxia lowers the oxygen supply and can rapidly reduce cytosolic pH by at least 0.5 pH units (Felle & Bertl, 1986; Fox et al., 1995; Guern et al., 1991; Muhling et al., 1995; Roberts et al., 1984; Sanders & Slayman, 1982). The reason for this plant organ acidification has been ascribed to lactic acid accumulation due to a metabolic shift to alcoholic fermentation (Roberts et al., 1984).

Plant pH variation is predominantly determined by the overall inherent differences among species or functional groups, rather than by the phenotypic variation of each species in response to differences in soil chemistry (Cornelissen et al., 2011; Liu et al., 2019). For example, a relatively stable leaf pH has been found in temperate herbaceous species grown in a broad range of at least four pH units (Liu et al., 2019). Here, however, both soil pH (5.05–9.58) and root pH showed a relatively large span (4.58–8.60; Figure S2), suggesting that roots across alpine grassland ecosystems have a weak self‐regulation capacity of pH. A strong positive relationship between soil and root pH across all herbaceous species on the Tibetan Plateau was observed, inconsistent with the results of Cornelissen et al. (2011) and Liu et al. (2019) who found soil pH had no significant influence on leaf pH. The variation in root pH of four widely distributed genera – *Stipa*, *Kobresia*, *Leontopodium* and *Potentilla* – along the soil and climate gradients was further explored to determine the effect of soil pH on intraspecific variation (Figure S3). The results showed that soil pH is also an external factor that strongly affects the root pH of a given plant. First, in contrast to short‐term plot culture experiments (Cornelissen et al., 2011), our investigation was carried out under long‐term absorption of basic cations by alpine plants. Second, the lack of calcium‐ or magnesium‐rich soils (soil pH >7) found in previous studies (Cornelissen et al., 2011) resulted in an unevaluable pH response of herbaceous plants to alkaline soils. In particular, dicot herbs have been shown to take up and transport Ca and Mg partly passively through the apoplast and therefore may accumulate these base cations at rather high concentrations, particularly in base‐rich soils (Rorison & Robinson, 1984; Thompson et al., 1997). Notably, different basic elements had contrasting effects on root pH for different functional groups. The soil Ca concentration significantly increased the root pH of most functional groups, whereas the soil K concentration had the most significant effect on the pH of sedge roots. Sedges tend to grow in humid environments and have a high water demand (He et al., 2008) which can also be seen in the distribution range of this function type over the precipitation gradient on the Tibetan Plateau (Table 1). The root pH of sedges is closely related to soil K concentration, which may be because sedges need to improve their water use efficiency and drought resistance by regulating plant physiological characteristics such as osmotic regulation ability, stomatal movement and transpiration rate (Felle, 2001; Liu et al., 2000) through K absorption to cope with occasional water fluctuations.

Although significant relationships between plant pH and MAT have been identified in forest ecosystems in northern China (Liu et al., 2019), no significant linear regression was observed between root pH and MAT across all species in the Tibetan alpine grasslands. This could be in part due to the smaller MAT extent of alpine grasslands (−2.73 to 2.21°C for our data set compared with −6.10 to 12.19°C recorded by Liu et al. (2019)) and also due to MAT which may be a less important limiting factor controlling biological (vegetation distribution and resource utilisation patterns) and geochemical (nutrient cycling) processes in alpine grassland ecosystems than moisture (Hong et al., 2016; Yang et al., 2009).

Here, the root pH of alpine herbaceous plants showed a positive trend with increasing SR. Radiation changes may indirectly affect root pH by influencing plant nutrients, soil pH and water content (Fu et al., 2022; Sun et al., 2023; Xiao et al., 2023). Changes in light intensity are also a basic signal which is transferred to pH changes within both the cytosol and apoplast (Felle, 2001). The responses of the three non‐legume groups are consistent with the finding that a decrease in light intensity could result in rapid cytosolic acidification due to increased substrate concentrations (H^+^) and hyperpolarisation of the plasma membrane (Felle, 2001; Felle & Bertl, 1986).

## 5 CONCLUSIONS

Our study shows that the root pH of alpine herbaceous plants is driven by the combined influence of climate, soil and PFGs. PFGs accounted for 5.39% of the root pH variation, whereas climate and soil explained 11.15 and 24.94%, respectively. Grasses had the highest root pH values compared with sedges, legumes and forbs. Moisture status was a stronger regulator driving root pH variability than temperature along environmental gradients. This study found a strong positive relationship between soil and root pH across all herbaceous species on the Tibetan Plateau. Our study provides new insights into root functional traits and survival strategies of herbaceous plants in alpine ecosystems.

## AUTHOR CONTRIBUTIONS

**Lirong Zhao:** Data curation (equal); formal analysis (equal); investigation (equal); validation (equal); visualization (equal); writing – original draft (equal); writing – review and editing (equal). **Bo Pang:** Data curation (equal); investigation (equal); writing – review and editing (equal). **Jiangtao Hong:** Conceptualization (equal); data curation (equal); funding acquisition (equal); resources (equal); writing – original draft (equal). **Xingxing Ma:** Data curation (equal); writing – review and editing (equal). **Ziyin Du:** Data curation (equal); writing – review and editing (equal). **Xiaodan Wang:** Conceptualization (equal); data curation (equal); funding acquisition (equal); resources (equal); writing – review and editing (equal).

## FUNDING INFORMATION

This research was supported by the Second Tibetan Plateau Scientific Expedition and Research Program (2019QZKK0404), the National Natural Science Foundation of China (42271070), the Technology Major Project of Tibetan Autonomous Region of China (XZ202201ZD0005G01), the West Light Scholar of Chinese Academy of Sciences (xbzg‐zdsys‐202202) and the Science and Technology Research Program of Institute of Mountain Hazards and Environment, Chinese Academy of Sciences (IMHE‐ZDRW‐04).

## CONFLICT OF INTEREST STATEMENT

No potential conflict of interest was reported by the authors.

## Supporting information


File S1.

Data S1.

## Data Availability

All supplementary data required for the main conclusions in the paper are contained in the paper and supplementary materials.
