# Supplementary material for: Root pH variation of herbaceous plants among plant functional groups in response to climate and soil gradients on the Tibetan alpine grasslands
Source: Ecol Evol. 2024 Jul 21;14(7):e70060. doi: 10.1002/ece3.70060 (PMC11260881; doi:10.1002/ece3.70060)
Supplement: Supplementary file 2 — Data S1. [file ECE3-14-e70060-s001.docx]

Root pH variation of herbaceous plants among plant functional groups in response to climate and soil gradients on Tibetan alpine grasslands

**Supporting information**

**Table S1** Vegetation characteristics of sampled species

**Table S2** Correlation coefficient between environmental factors (climatic and soil factors).

**Figure S1** Distribution of sampling sites

**Figure S2** Frequency distribution of roots pH

**Figure S3** Relationship between soil pH and root pH for four widely distributed genus

Table S1 Vegetation characteristics of sampled species

| **Species** | **Family** | **Genus** | **Plant functional groups** | **Phylogeny** |
| --- | --- | --- | --- | --- |
| *Elymus nutans* | Gramineae | Elymus | Grass | Monocotyledon |
| *Poa litwinowiana* | Gramineae | Poa | Grass | Monocotyledon |
| *Stipa glareosa* | Gramineae | Stipa | Grass | Monocotyledon |
| *Stipa purpurea* | Gramineae | Stipa | Grass | Monocotyledon |
| *Kobresia humilis* | Cyperaceae | Kobresia | Sedge | Monocotyledon |
| *Kobresia littledalei* | Cyperaceae | Kobresia | Sedge | Monocotyledon |
| *Kobresia pygmaea* | Cyperaceae | Kobresia | Sedge | Monocotyledon |
| *Carex moorcroftii* | Cyperaceae | Carex | Sedge | Monocotyledon |
| *Astragalus confertus* | Leguminosae | Astragalus | Legume | Dicotyledon |
| *Oxytropis glacialis* | Leguminosae | Oxytropis | Legume | Dicotyledon |
| *Oxytropis microphylla* | Leguminosae | Oxytropis | Legume | Dicotyledon |
| *Saussurea tibetica* | Compositae | Saussurea | Forb | Dicotyledon |
| *Saussurea leontodontoides* | Compositae | Saussurea | Forb | Dicotyledon |
| *Christolea crassifolia* | Cruciferae | Christolea | Forb | Dicotyledon |
| *Artemisia wellbyi* | Compositae | Artemisia | Forb | Dicotyledon |
| *Artemisia demissa* | Compositae | Artemisia | Forb | Dicotyledon |
| *Leontopodium nanum* | Compositae | Leontopodium | Forb | Dicotyledon |
| *Suaeda glauca* | Chenopodiaceae | Suaeda | Forb | Dicotyledon |
| *Chenopodium tibeticum* | Chenopodiaceae | Chenopodium | Forb | Dicotyledon |
| *Polygonum capitatum* | Polygonaceae | Polygonum | Forb | Dicotyledon |
| *Ceratoides latens* | Chenopodiaceae | Ceratoides | Forb | Dicotyledon |
| *Potentilla bifurca* | Rosaceae | Potentilla | Forb | Dicotyledon |
| *Ajania fruticulosa* | Compositae | Ajania | Forb | Dicotyledon |
| *Ptilotricum canescens* | Cruciferae | Ptilotricum | Forb | Dicotyledon |
| *Aster hispidus* | Compositae | Aster | Forb | Dicotyledon |

Table S2 Correlation coefficient between environmental factors (climatic and soil factors).

|  | MAP (mm) | MAT(^o^C) | AI (unitless) | SR (MJ m^-2^ year^-1^) | K_soil_ (%) | Soil pH(unitless) | Soil water (%) | CEC (cmol kg^-1^) | Soil Ca (mg g^-1^) | Soil K (mg g^-1^) | Soil Mg (mg g^-1^) | Soil Na (mg g^-1^) |
| --- | --- | --- | --- | --- | --- | --- | --- | --- | --- | --- | --- | --- |
| MAP (mm) | 1 |  |  |  |  |  |  |  |  |  |  |  |
| MAT (^o^C) | 0.058 | 1 |  |  |  |  |  |  |  |  |  |  |
| AI (unitless) | 0.943*** | -0.020 | 1 |  |  |  |  |  |  |  |  |  |
| SR (MJ m^-2^ year^-1^) | -0.813*** | 0.061 | -0.895*** | 1 |  |  |  |  |  |  |  |  |
| K_soil_ (%) | 0.887*** | 0.046 | 0.842*** | -0.643** | 1 |  |  |  |  |  |  |  |
| Soil pH(unitless) | -0.829*** | 0.152 | -0.844*** | 0.914*** | -0.647** | 1 |  |  |  |  |  |  |
| Soil water (%) | 0.708*** | -0.128 | 0.781*** | -0.915*** | 0.545* | -0.943*** | 1 |  |  |  |  |  |
| CEC (cmol kg^-1^) | 0.647** | -0.247 | 0.603** | -0.633** | 0.584** | -0.733*** | 0.726*** | 1 |  |  |  |  |
| Soil Ca (mg g^-1^) | -0.538* | 0.217 | -0.469* | 0.284 | -0.462* | 0.439 | -0.308 | -0.141 | 1 |  |  |  |
| Soil K (mg g^-1^) | -0.213 | -0.160 | -0.261 | 0.351 | -0.110 | 0.291 | -0.345 | -0.371 | -0.403 | 1 |  |  |
| Soil Mg (mg g^-1^) | -0.389 | -0.113 | -0.300 | 0.212 | -0.450* | 0.374 | -0.241 | -0.062 | 0.670** | -0.339 | 1 |  |
| Soil Na (mg g^-1^) | -0.680** | -0.083 | -0.553* | 0.493* | -0.675** | 0.540* | -0.446* | -0.680** | 0.064 | 0.502* | 0.101 | 1 |

*Note*. **P*<0.05, ***P*<0.01, ****P*<0.001.


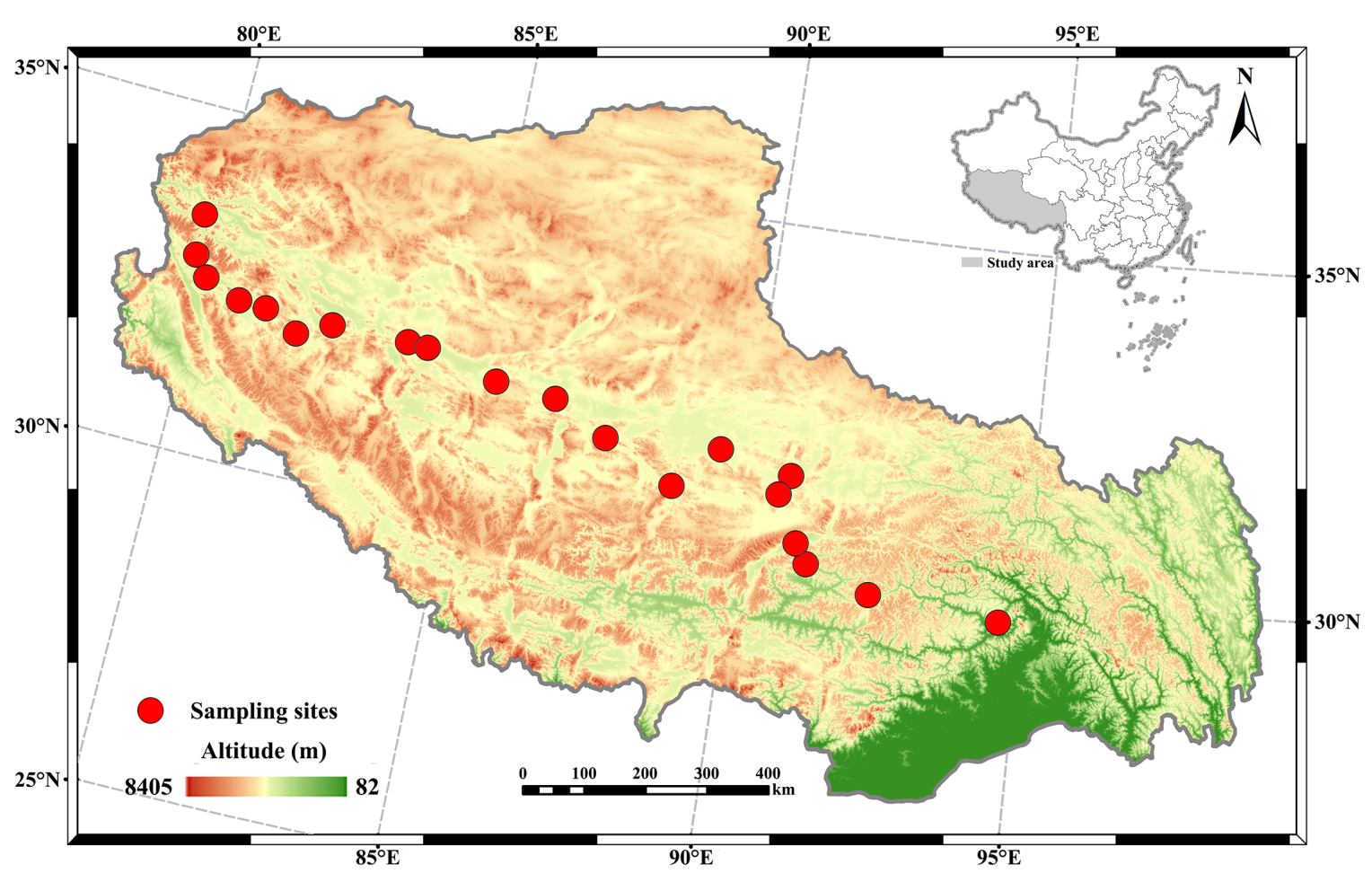


Figure S1 Distribution of sampling sites





Figure S2 Frequency distribution of roots pH





Figure S3 Relationship between soil pH and root pH for four widely distributed genus
